# Early-Life Nutritional Factors and Mucosal Immunity in the Development of Autoimmune Diabetes

**DOI:** 10.3389/fimmu.2017.01219

**Published:** 2017-09-28

**Authors:** Ling Xiao, Belinda van’t Land, Wouter R. P. H. van de Worp, Bernd Stahl, Gert Folkerts, Johan Garssen

**Affiliations:** ^1^Faculty of Science, Utrecht Institute for Pharmaceutical Sciences, Utrecht University, Utrecht, Netherlands; ^2^Nutricia Research, Utrecht, Netherlands; ^3^Department of Pediatric Immunology, Wilhelmina Children’s Hospital, University Medical Centre Utrecht, Utrecht, Netherlands

**Keywords:** type 1 diabetes, gut microbiota, mucosal immunity, tolerogenic dendritic cells, short-chain fatty acids, human milk oligosaccharides, nutrition

## Abstract

Type 1 diabetes (T1D) is an immune-mediated disease with a strong genetic basis but might be influenced by non-genetic factors such as microbiome development that “programs” the immune system during early life as well. Factors influencing pathogenesis, including a leaky intestinal mucosal barrier, an aberrant gut microbiota composition, and altered immune responsiveness, offer potential targets for prevention and/or treatment of T1D through nutritional or pharmacologic means. In this review, nutritional approaches during early life in order to protect against T1D development have been discussed. The critical role of tolerogenic dendritic cells in central and peripheral tolerance has been emphasized. In addition, since the gut microbiota affects the development of T1D through short-chain fatty acid (SCFA)-dependent mechanisms, we hypothesize that nutritional intervention boosting SCFA production may be used as a novel prevention strategy. Current retrospective evidence has suggested that exclusive and prolonged breastfeeding might play a protective role against the development of T1D. The beneficial properties of human milk are possibly attributed to its bioactive components such as unique immune-modulatory components human milk oligosaccharides and metabolites derived thereof, including SCFAs. These components might play a key role in healthy immune development and creating a fit and resilient immune system in early and later life.

## Introduction

Type 1 diabetes (T1D) is one of the most common autoimmune diseases, which develop during infancy and results in T-cell-mediated destruction of β-cells within the pancreas in especially genetically susceptible individuals (1). T1D incidence is rising significantly (relative annual increase in incidence is estimated to be 1.8%) according to the most recent multicenter observational study (2). Because genetic manifestation is constant, environmental factors such as infections, intestinal microbiota, vaccines, hygiene, and dietary factors (e.g., breastfeeding, cow’s milk, solid food and cereals, vitamin D3) have been suggested to influence the development of T1D in genetically predisposed populations (3). Therefore, it is important to understand the role of these environmental factors with the aim to find new possibilities to reduce the increase in incidence and finally prevent or delay the development of T1D. In this review, the potential role of human milk has been evaluated and possible new strategies using bioactive components from human milk to prevent T1D development discussed.

The involvement of mucosal immunity (including microbial composition, intestinal lining, and mucosal immune cells) has been emphasized in the etiology of T1D (4). This is shown by the immunological link between the gastrointestinal tract (GI tract) and the pancreas [i.e., T-cells activated in the intestine can migrate into the pancreatic islets (5)], highlighting the GI tract as a potential target for intervention. Therefore, it is plausible that a strategy which induces an optimal establishment of the intestinal microbiota and ensuing proper immune maturation early in life might be essential for protection against T1D development (6).

One of the first factors in life establishing a stable mucosal immune development is human milk. In the postnatal developing gut, human milk is thought to suppress inflammatory processes and reduce the risk to develop multiple immune-mediated diseases such as autoimmune diabetes, allergic diseases, and infectious diseases (7, 8). Furthermore, human milk contains multiple functional compounds with immunological properties, including but not limited to human milk oligosaccharides (HMOS) (9). After consumption of non-digested fibers such as HMOS, the production of short-chain fatty acids (SCFAs) is increased. Interestingly, specific SCFAs have recently been demonstrated to protect against T1D within murine diabetes models (10). The complex mixture of glycans within human milk is known to have specific prebiotic, anti-adhesive, antimicrobial, and immune-modulatory properties (11). They are abundantly present in human milk and are believed to contribute to the health benefits of human milk. However, so far little is known about their potential to protect against T1D. In this review, dietary interventions with SCFAs and HMOS have been discussed as examples of environmental factor-induced complex interplay between intestinal microbiota, intestinal barrier function, and immunity in the development of T1D.

## Pathogenesis of T1D

### Molecular Pathogenic Mechanisms of T1D

**Figure 1:**
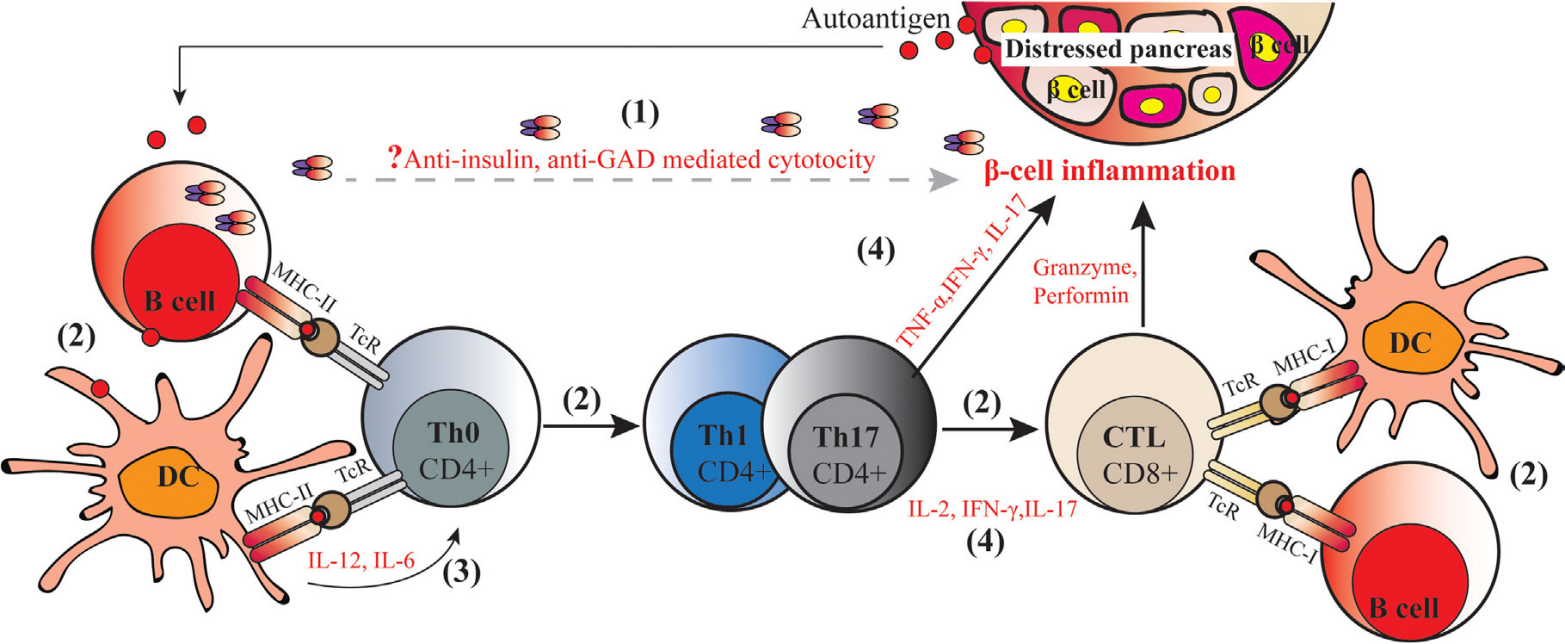
Molecular pathogenic mechanisms of type 1 diabetes (T1D). The autoimmune process of T1D includes the following: (1) secretion of autoantibodies against islet proteins such as insulin and glutamic acid decarboxylase (GAD) by autoreactive B cells, which are viewed as markers but have not been assigned a direct pathogenic role on the development of diabetes; (2) autoantigens capture and (cross-) present to naïve CD4^+^ and CD8^+^ T cells by autoreactive antigen-presenting cells including macrophages, dendritic cells (DCs), and B cells through two signals, namely, presentation of autoantigens in MHC–peptide and activating co-stimulatory molecules, leading to induction of autoreactive helper CD4^+^ T cells, and cytotoxic CD8^+^ T cells; (3) production of (pro-)inflammatory cytokines by DCs that stimulate Th0 to convert into Th1 and Th17 cells; (4) secretion of inflammatory cytokines by Th1 and Th17 cells trigger CD8^+^ T cells to secret cytotoxic granzyme, perforin, nitric acid, peroxide, and other inflammatory cytokines. In this way, CD4^+^ T cells and CD8^+^ T cells act synergistically to kill islet β-cell in conjunction with β-cell autoantigens and MHC class I and class II antigens, resulting in the onset of autoimmune diabetes.

Type 1 diabetes is an autoimmune disorder that involves several components of the immune system negatively interacting with pancreatic islets. Initially β-cell proteins [e.g., insulin, glutamic acid decarboxylase, and protein tyrosine phosphatase (IA2 or ICA512)] are exposed for instance due to viral infection on the islet, to the immune system. Antigen-presenting cells (APC), especially dendritic cells (DCs), process and present autoantigens to naïve CD4^+^ T cells (Th0). Presentation through major histocompatibility complex class II (MHC-II) molecules and co-stimulatory molecules leads to activation of auto reactive CD4^+^ T cells. Cross-presentation of processed autoantigens through MHC-I stimulate CD8^+^ T cells activation. At the same time, auto reactive DCs secrete pro-inflammatory cytokines such as IL-12 and IL-6 which stimulate the naïve Th0 cells to convert to autoreactive Th1 and Th17 cells after processing the autoantigen. Expanded autoreactive CD4^+^ T cells secrete inflammatory cytokines such as IFN-γ, IL-2, and IL-17 which may in turn trigger cytotoxic lymphocytes (CTL) to stimulate pancreatic islet inflammation (insulitis) and/or directly kill pancreatic islet β-cells; all these processes together contribute to T1D onset and progression (depicted in Figure 1).

In the past decade, the critical role of CD4^+^CD25^+^ regulatory T cells (Tregs) deficiency in the pathogenesis of autoimmunity has been emphasized. Tregs seem to bridge the central and peripheral processes: central tolerance to autoantigens involve clonal depletion of autoreactive T cells in the thymus during development; in case of incomplete deletion or escaping central tolerance through, e.g., posttranslational modification mechanisms, which contributes to generation of novel autoantigens such as modified pre-pro-insulin. While the deletion of autoreactive T cells within the thymus and the induction of anergy in the periphery represent major mechanisms in the maintenance of immune fitness, Tregs are thought be an additional mechanism to avert autoimmunity. Tregs are widely demonstrated to be capable of suppressing proliferation and inflammatory cytokine production of autoreactive CD4^+^ and CD8^+^ T cells thus are pivotal for the maintenance of peripheral tolerance. In T1D patients, deficiency in suppressor function, but not frequency of Tregs, has been shown to contribute to the loss of tolerance to beta-cell antigens (12). Therefore, a therapeutic approach inducing and expanding Tregs involved in autoimmune regulation may restore tolerance in diabetes patients.

A disrupted immune homeostasis can cause prolonged inflammation or immune activation as a consequence of the intense interplay between the outside world (including food antigens, commensal and pathogenic microbes) and the mucosal immune system (6). Many recent discoveries associate T1D development with an abnormal deregulated intestinal immune system implying (1) an altered gut microbiota composition, (2) loss of intestinal integrity, referred to as “leaky gut,” and (3) an altered mucosal immunity. This section will focus on the involvement of these three facets in the development of T1D.

### Altered Gut Microbiota Composition in T1D

Given the intimate interplay between the microbial community at different bodily sites, such as the oral cavity, gut, skin, lung, and immune system, it is not surprising that some members of the commensal microbiota have been linked to the increasing development of autoimmune diseases. The first evidence within T1D arrived from studies in Bio Breeding Diabetes Prone (BB-DP) rats and non-obese diabetic (NOD) mice, where the administration of antibiotics decreased incidence of T1D (13, 14). Moreover, specific pathogen-free (SPF) NOD mice lacking MyD88, an adaptor protein for innate immune toll-like receptors (TLRs) that recognize microbial stimuli, were protected from T1D and showed a different microbial composition compared to the wild-type controls. By contrast, germ-free (GF) NOD mice lacking the MyD88 protein robustly developed T1D (15). Data from this study provide the first clear interactions between the intestinal microbiota and the innate immune system. Altogether, these results suggest the critical role the gut microbiota play in the development of T1D.

Due to developments in next generation sequencing technologies, detailed studies on the role of specific (gut) microbiota composition in development of autoimmune disease are more accessible. Indeed, various studies have demonstrated that specific (combinations of) bacteria groups are correlated with T1D disease development. First of all, though non-diabetic children are able to build a healthy and stable microbiome, the microbiomes of diabetic children are less diverse and more unstable in nature (16). It is believed that gut microbiota with limited diversity may reduce the capacity to digest a diverse diet, resulting in reduced energy intake in affected individuals. Second, the ratio of *Bacteroidetes* to *Firmicutes* seems to be a hall-mark within T1D. Within small observational studies (like the Finish cohort analysis), the microbiome showed a decline in *Firmicutes* and increase in *Bacteroidetes* over time as these children became autoimmune, while the opposite was seen within age and gender matched healthy controls (16). In addition, it is believed that butyrate-producing bacteria and mucin degraders play a protective role in T1D. A metagenomic analyses of fecal samples showed that many of the bacterial genera significantly less abundant in T1D compared to controls, are the butyrate producers such as *Faecalibacterium* and *Subdoligranulum*, and mucin degraders such as *Prevotella* and *Akkermansia*. By contrast, producers of other SCFAs, such as *Bacteroides, Veillonella*, and *Alistipes* were more abundant within diabetic individuals (17). Consistent with results of these previous studies, a more recent study conducted in a Spanish cohort of children diagnosed with T1D and healthy controls also showed an increase in *Bacteroidetes* and a decrease in *Firmicutes*. Furthermore, authors from this study found a negative correlation between the number of *Bifidobacterium* and *Lactobacillus* and the plasma glucose level, while the number of *Clostridium* was positively correlated with the plasma glucose level in the diabetes group (18). Although the outcomes of both small studies show some discrepancy, the results illustrate interesting observations regarding changes within microbiome composition. More studies with bigger cohorts are needed to validate the impact of environmental influences on the altered T1D microbiome. Possible mechanisms that can explain the inter-relationship between specific bacterial genotypes with a healthy infant and an infant with T1D are discussed below.

Collectively, data from these published studies suggest that low diversity and reduced stability of gut microbiota, increased ratio of *Bacteroidetes* versus *Firmicutes*, and a low abundance of butyrate-producing bacteria are somehow associated with β-cell autoimmunity and T1D development. The alterations in gut microbiota composition could lead to aberrancies in the intestinal mucosal immune system, such as increased gut permeability, small intestinal inflammation, and loss of tolerance to food antigens, all of which are associated with the development of T1D.

### Leaky Gut in T1D

The intestinal epithelial cells form a permeable luminal lining of the GI tract and are important in the absorption of nutrients and the protection against harmful substances. Tight junctions (TJs) are proteins of the intestinal epithelium regulating barrier integrity. The TJ complexes, formed between the apical and basolateral sites of the epithelial cells, are primarily composed of the Claudin and Occludin protein family (19). Increased intestinal permeability can lead to increased para-cellular transport of harmful substances contributing to intestinal inflammation, as well as activation of the diabetogenic CD8^+^ T cells, which in turn lead to progression of insulitis. This notion is supported by evidence from both experimental animal models and human studies. In BB rats, an increased gut permeability was observed in early onset of T1D (20). The increased gut permeability in BB rats was associated with structural changes in intestinal morphology and decreased expression of TJ proteins Claudin-1 and Occludin (20). In addition, increased gut permeability was observed before the onset of T1D in NOD mice as well (18). Moreover, infection of the intestinal epithelium with *C. rodentum* in 4-week-old NOD mice (leading to interrupted intestinal barrier) stimulated the development of T1D. After administration, *C. rodentum* was localized in the mesenteric lymph nodes (MLN) and in the pancreatic lymph nodes (PLN) of the infected mice. Moreover, proliferation of CD8^+^ cells and activation of polyclonal T-cells was observed in the PLN. Collectively, these results from animal studies suggest that intestinal permeability plays a pathogenic role in the onset and development of T1D and intestinal infection caused loss of barrier function may contribute to a loss of pancreatic islets β-cells and insulitis in animal models (21). In humans, several studies demonstrate that the intestinal permeability is increased in people predisposed or already diagnosed with T1D established by sugar permeability tests (22, 23).

Additionally, alterations in gut microbiota composition might contribute to gut permeability changes in T1D as well. As described in the previous paragraph, children prone to develop or diagnosed with T1D show a decrease in butyrate-producing bacteria (17). Butyrate plays a role in the maintenance of gut integrity through the induction of mucin synthesis and increased TJ production (17, 24). By contrast, bacterial genera that produce other SCFAs, such as acetate, propionate, and succinate, are increased in T1D patients. However, these SCFAs do not induce mucin synthesis and TJ production (24, 25). This information shows the complex interplay between the different factors and the importance to tackle all of them in the treatment or prevention of T1D.

### Altered Intestinal Immunity in T1D

The interaction of microbiota with the innate immune system provides signals to promote the maturation of immune cells and the normal development of immune functions, i.e., immune fitness (26). Antigen-presenting DCs, resident in the lamina propria (LP), sample luminal antigens and present them to T-cells. This function of DCs is essential in developing the sensitive balance between immune activation and tolerance (27). In the case of T1D, it is the loss of tolerance to self that leads to autoimmunity (28). In relation to the importance of a balanced host microbe interaction, the mucosal immune system may be skewed toward immune activation. Indeed, this has been shown from small intestinal biopsies of the intestines from children with T1D, which were analyzed for level of immune activation. Children with T1D show enhanced expression of HLA-II molecules, increased expression of ICAM-1 on epithelial cells, and α4β7-integrin on cells of the LP. In addition, increased numbers of IL-4, IL-1α, and IFN-γ expressing immune cells were reported, suggesting overall increased mucosal immune activation (29). As discussed above, deficiency of Tregs contributes to the molecular mechanism of T1D. It was demonstrated that the number of Forkhead box protein 3 (Foxp3)-positive Tregs was low and did not show activation of FoxP3 transcripts in small intestinal biopsies from children with T1D, supporting a defect in the intestine within this regulatory mechanism (30). Another study confirmed the absence of active FoxP3^+^ Tregs in the small intestine and demonstrated that LP-derived DCs of T1D patients were not even capable of inducing FoxP3^+^ Tregs *in vitro* possibly leading to an over-activated mucosal immunity within T1D (31). In correlation, it has been reported that children with T1D have an increased number of activated IL-17 secreting CD8^+^ and CD4^+^ T-cells, likely due to lack of suppressive mechanisms (32). Moreover, neutralizing this pro-inflammatory IL-17 has been associated with a reduced insulitis score in the effector phase of disease development in NOD mice, suggesting a role for this cytokine and immune activation within pathogenesis of T1D (33).

These findings support the hypothesis that abnormal activation of the mucosal immune system is involved in the pathogenesis of T1D. Knowing the three important components for the pathogenesis of T1D, it seems plausible that intervention with nutritional or pharmacologic means targeted to maintain a non-diabetogenic microbiota composition, properly regulate the intra-epithelia TJ, as well as prevent excessive mucosal inflammation and rebalance autoimmune responses represents a clear potential for the prevention and treatment of T1D.

## The Importance of Tolerogenic Dendritic Cells (tDCs) in the Development of Autoimmunity

The GI tract is comprised of a huge amount of immune cells to achieve a state of immune homeostasis and tolerance, i.e., immune fitness. Particularly, being the ultimate gatekeepers of the immune responses, DCs play a critical role in controlling effector and regulatory mechanisms relevant to the pathology of autoimmune diseases. Immature DCs residing in the peripheral tissues induce anergy and secretion of immunomodulatory cytokines to maintain tolerogenicity and avoid destructive T cell auto-reactivity. On the other hand, these DCs mature upon sensing various danger signals such as autoantigens and may stimulate conversion of autoreactive T cells from naïve T cells thus losing their regulatory competences. Therefore, adoption of tolerogenic phenotype of DCs is important for the induction of immunogenic tolerance. Recent reviews have suggested that the induction of a tolerogenic phenotype of DCs provides a novel antigen-specific strategy for the prevention and treatment of autoimmunity (34–36).

### tDCs Mechanisms of Action

**Figure 2:**
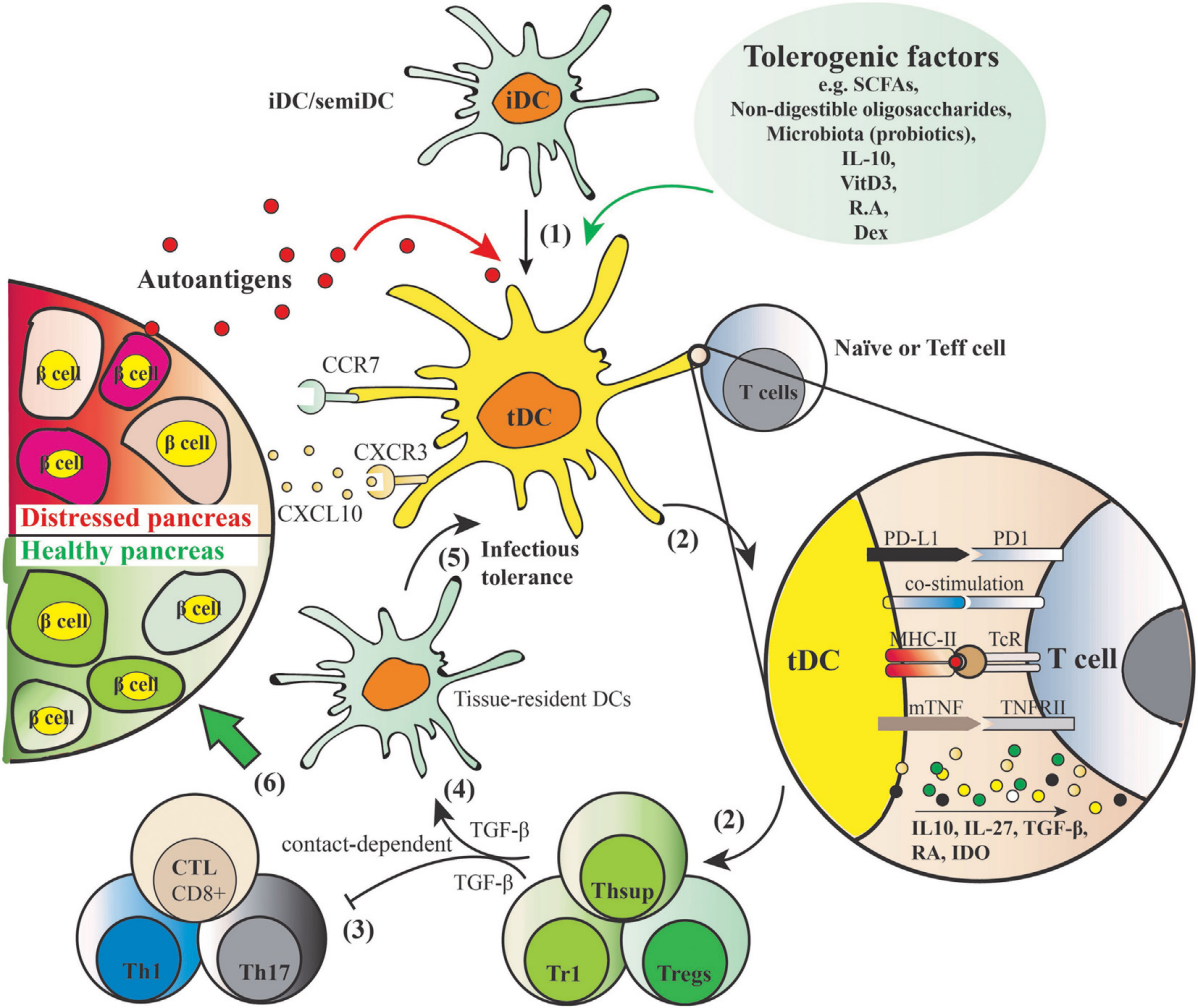
Induction of autoantigen-specific tolerance by tolerogenic dendritic cells (tDCs) and mechanism of action. (1) Tolerogenic factors such as short-chain fatty acids, non-digestible oligosaccharides, microbiota, IL-10, vitamin D3, retinoic acid, and dexamethasone induce a tolerogenic phenotype of dendritic cells (DCs) with shared features include resistance of maturation, lowered levels of co-stimulatory markers (e.g., CD40, CD80, CD83, CD86), elevated levels of programmed death ligand 1 (PD-L1) [PD-L1–PD1 signaling induces apoptosis of effector Th1 and cytotoxic lymphocytes (CTL), and is crucial for acquisition of regulatory T cell (Treg) function] and membrane bound TNF, induction of anti-inflammatory cytokine profiles (low IL-12 and IL-6, IL-10, IL-27, and TGF-β), and increased expression of migration markers such as CXCR3 and CCR7 that allows for migration to the lymphoid organs and inflammatory lesion in the pancreas producing CXCL10. (2) When these tDCs are pulsed with autoantigens and exposed to cognate naïve or effector T cells, they present processed autoantigens to the T cells to induce Tregs. (3) Natural or induced Tregs suppress the activation of autoreactive Th1/Th17 and cytotoxic CD8^+^ T cells in a cell contact–dependent manner. (4) In addition, inhibitory mediators such as TGF-β produced by the induced secondary T suppressor cells (Thsup) inhibit the activation of T effector cells. (5) Convert the tissue-resident DCs to tDCs through an “infectious tolerance” mechanism, thereby reinforcing the tolerogenic phenotype. This secondary systemic suppressive effect is cell contact independent. (6) These five mechanisms mediated by tDCs together contribute to the desired endpoint, namely, a healthy pancreas.

Dendritic cells play a key role in both central and peripheral tolerance mechanisms. During central tolerance, iDCs promote immune tolerance by negatively selecting autoreactive T-cells in the thymus. Peripherally, tDCs function through the induction and stimulation of antigen-specific Tregs. As depicted in Figure 2, iDCs or semi-mature DCs adopt tolerogenic phenotype by being incubated with, or differentiated in, the presence of various tolerogenic factors (listed in Figure 2). When these induced tDCs are pulsed with autoantigens such as insulin in the context of autoimmune diabetes, and upon cognate interaction with naïve T cells or effector T cells, they present their processed autoantigen peptides to the T cells and instruct and stimulate antigen-specific Tregs. At the same time, tDCs induce tolerance in a contact-independent manner by secreting cytokines such as TGF-β. Subsequently, those induced Tregs through infectious tolerance mechanisms can transfer their tolerogenic potencies to pro-inflammatory DCs by stimulating the expression of regulatory receptors (37), as well as to another T cell population with a different antigen specificity (38), thus contributing to reinforced tolerance against specific autoantigens. Finally, tDCs are equipped with suitable migration ability which may allow them to elicit local inhibitory effects in the inflamed pancreas.

### Maturation Status, Tolerogenic Phenotypes, and Migration Capacity of tDCs

It has been suggested that the regulatory function of DCs is determined by their activation status as well as the cytokine microenvironment. Multiple studies show that DCs have regulatory properties in their immature state (39, 40). However, increasing evidence indicates that under adequate conditions fully matured DCs can induce tolerance as well (41, 42). This suggests that also other, more complex, factors such as DC lineage, cell surface antigens, and cytokine milieu determine whether DCs can elicit tolerogenic activity and support the differentiation of Tregs. Both programmed death ligand 1 (PD-L1) and membrane-bound TNF (mTNF) play an important role in this process (43, 44). PD-L1 expressed on tDCs may shut down autoreactive T cells in the periphery through inducing Tregs. On DCs modulated by vitamin D3, PD-L1 has been shown to be crucial for the acquisition of Tregs function by CD4^+^ T cells, and blockage of PD-L1–PD1 signaling during T-cell priming results in the generation of Th1 incapable of suppressing T-cell proliferation and producing IL-10 (43). Besides, it has been suggested that PD-L1 expressed particularly in the islet is involved in inducing apoptosis of effector T cells (CTL and Th1) (45). mTNF also been shown to be essential for the induction of antigen-specific Tregs by vitamin D3 modulated DCs (44), and blockage of mTNF–TNFRII interaction during Treg induction abrogated their suppressive function. Similarly, the binding of CD80/CD86 with CTLA-4 on T-cells, but not CD28, forces Treg differentiation (46).

Different subsets of DCs have specific tolerogenic properties in certain tissue-specific LNs. A population of intestinal DCs expressing the surface antigen CD103 has been shown to be crucial in inducing tolerance to commensal bacteria and other (e.g., food) antigens. Gut-associated DCs include those in the LP of the intestine and the gut-associated lymphoid tissue, which includes the isolated lymphoid follicles, the Peyer’s patches, and the MLN. Almost all the LP-DCs and a subset of MLN DCs express CD103. These CD103^+^ DCs are crucial for the gut homeostasis through different mechanisms. Under steady-state conditions, they induce tolerance through production of different suppressive cytokines or beneficial dietary metabolites when facing discriminating challenge of pathogens. CD103^+^ DCs, but not CD103^−^ DCs in the intestinal LP promote the differentiation of FoxP3^+^ Tregs in a TGF-β- and retinoic acid (RA)-dependent manner (47, 48). Consistently, CD103^+^ DCs in the intestinal MLN were shown to induce the development of FoxP3^+^ Tregs through the same mechanism (49). These findings together suggest induction of intestinal CD103^+^ DCs as a potent strategy for the prevention or treatment for autoimmune diabetes. Surprisingly, the presence of CD103^+^ DCs in the PLN play a detrimental role for the development of autoimmune diabetes in the NOD.*Batf3*^−/−^ mouse model (50). By contrast, another subset of DCs existing in pancreatic LN, namely, the CD11c^+^CD11b^+^ DCs, has been demonstrated to have a tolerogenic role in autoimmune diabetes (51). Transfer of these CD11c^+^CD11b^+^ DCs that express low levels of CD40, MHC-II, and CD80/86 suppressed the onset of autoimmune diabetes in an *in vivo* NOD mouse model in a CCL2/CCR2 signaling-dependent manner (51), suggesting that induction of this type of DC in specific tissue LNs, namely, pancreatic LNs has potential for the protection against T cell-mediated autoimmune destruction of pancreatic islets. Furthermore, this subset of DCs has also been shown to play a major role in intravenous tolerance and the suppression of experimental autoimmune encephalomyelitis (52). Finally, the expression of suitable migration markers such as CCR7, induced by tolerogenic factors such as vitamin D3 or dexamethasone (43), may enable tDCs to migrate to lymphoid organs, where conversion of naive T cells into antigen-specific Tregs will occur, while CXCR3 expression may facilitate migration to the inflamed or destructive lesion in the pancreas to counteract autoreactive T cells.

### Cytokine Profiles Induced by tDCs and Autoimmunity

Next to the inhibitory surface markers, proper polarizing mediators (e.g., higher levels of IL-10, IL-27, TGF-β, RA, IDO, lower levels of IL-6 and IL-12) produced by tDCs activated by countersignaling from engaged T cells also contribute to Tregs instruction. IL-10 is an anti-inflammatory cytokine acting on both iDCs and (regulatory) T-cells (53). Maturation of DCs in the presence of IL-10 has been shown to reduce the expression of surface markers such as CD83 and increase the expression of inhibitory receptors such as PD-L1 (35, 36). Particularly, IL-10 may drive differentiation of IL-10-producing type-1 regulatory T cells (Tr1) but not FoxP3-positive CD4^+^CD25^+^ Treg cells (54). Next to IL-10, IL-27 signaling in DCs has been shown to limit the differentiation of effector T-cells and the development of EAE, but promote FoxP3^+^ Tregs and Tr1 expansion (55). RA produced by tDCs has also been shown to promote Tregs and control Th17 cells differentiation (47, 56). In the presence of MLN CD103^+^ DCs, TGF-β has the ability to enhance the conversion of naïve T cells into FoxP3^+^ Tregs; interestingly, RA is suggested to be essential for Tregs generation (49). tDCs induced by dexamethasone and vitamin D3 have been shown to inhibit the destructive immune response in a rheumatoid arthritis mouse model, and expression of TGF-β1 was significantly higher in these tDCs than mature DCs (57). Importantly, by inhibiting TGF-β1 signaling, these tDCs seems to regulate CD4^+^ T cells from rheumatoid arthritis patients in a TGF-β1-dependent manner. Interestingly, both IL-10 and IL-27 have a role in promoting tDCs development (53, 55). Other factors including SCFAs (58), synthetic non-digestible oligosaccharides (59), vitamin D3, corticosteroids, rapamycin, dexamethasone, and neuropeptides [summarized in a recent review (60)] have been shown to induce tDCs. These possibilities of generating tDCs open new therapeutic and maybe even preventive perspectives in autoimmune diseases and therefore also in T1D development.

## SCFAs may Protect Against T1D

Short-chain fatty acids are bacterial metabolites produced in the colon after bacterial fermentation of non-digestible dietary carbohydrates, including HMOS. SCFAs, mainly acetate, propionate, and butyrate, are known to form a communication link between the microbiota and the immune system by several means, including modulation of gut integrity (mostly through influencing the intestinal epithelial cells) (20), development of DCs and Tregs, induction of (anti)-inflammatory effects through signaling pathways such as activating G-protein-coupled receptors (GPCRs), inhibiting histone deacetylase (HDAC), stimulation of histone acetyl transferase activity, and stabilizing hypoxia inducible factor (HIF) (61, 62). Moreover, SCFAs can directly shape the pancreatic immune environment and autoimmune diabetes development (63). Evidence showing the direct protective effects of SCFAs against T1D (from *in vitro* as well as from animal models) and proposed mechanisms are reviewed in this section.

### SCFAs Maintain Gut Integrity

As mentioned before, a damaged mucosal integrity in the setting of T1D would allow for greater exposure of the intestinal immune system to antigens, which trigger the onset of T1D. This suggests that modulators of TJs may play a role in modulation of “intestinal leakiness.” The presence of luminal SCFAs, mainly butyrate, have been shown to be crucial in regulating the epithelial barrier function. Butyrate induces mucin synthesis [whereas propionate and acetate do not (17, 25)], decreases bacterial transport across metabolically stressed epithelia, and improves the intestinal barrier by increasing TJ assembly (17). Mucin is well known as a glycoprotein produced by the host that is believed to contribute to gut integrity (17, 24). In addition, butyrate enhances the barrier function by regulating the assembly of the TJ molecules ZO-1 and Occludin *via* the activation of AMPK (64). HIF is a transcription factor directly involved in maintaining gut integrity by regulating the production of antimicrobial peptides and epithelial TJs. Recently, it has been demonstrated that SCFAs indirectly stabilize HIF in intestinal epithelial cells (62). These findings support that microbial-induced butyrate production, and subsequent mucin synthesis, with a corresponding enhancement of TJ may contribute to the development of autoimmunity for T1D.

### SCFAs Modulate Innate and Adaptive Immunity

Next to the effects of SCFAs on maintaining gut integrity, several studies have shown that SCFAs play a pivotal role in promoting (mucosal) immune homeostasis and health *via* their direct immune-modulatory effects on immune cells such as (intestinal) DCs and intestinal epithelial cells. *In vitro*, butyrate and propionate, but not acetate, have been shown to directly induce a semi-mature phenotype and an anti-inflammatory gene expression profile on human monocyte-derived DCs after 24 h incubation. Consistently, both butyrate and propionate significantly reduced pro-inflammatory cytokine IL-6 and suppressed LPS-induced IL12p40 production (65). This suggests that both butyrate and propionate are promising in maintaining immune homeostasis by reducing the pro-inflammatory Th1 and Th17 phenotypes. Another *in vitro* study demonstrated that DCs stimulated with butyrate induced the expression of the immunosuppressive enzyme indoleamine 2,3-dioxygenase 1 and aldehyde dehydrogenase 1 A2 (66). The SCFA-dependent induction of indoleamine 2,3-dioxygenase 1 potentiates the ability of DCs to convert naïve T cells into FoxP3^+^ Tregs and consequent ability to suppress naïve T cells into IFN-γ^+^ T cells and/or Th17 cells. Moreover, the plasma membrane transporter SLC5A8, which is a high-affinity transporter for SCFAs, seems obligatory for the tolerogenic phenotype of DCs. These findings have implications for the role of SCFA in autoimmune disease development. As discussed above, intestinal DCs (particularly MLN DCs) expressing CD103 play an important role for the maintenance of mucosal homeostasis therefore may be an important target for the intervention of T1D (49). SCFAs derived from fiber-enriched diets have been shown by recent study to induce CD103^+^ tDCs in MLN through promoting RA production of intestinal epithelial cells, promoting the differentiation of FoxP3^+^ Tregs from naïve T cells (58). Interestingly, it has been shown that addition of SCFAs in the drinking water directly increased the number of FoxP3^+^ Tregs and IL-10^+^FoxP3^+^ Tregs in the colon of SPF mice, this effect has been suggested to be dependent on GPCR43 activation by SCFAs (67). In T1D, inflammation contributes to the early induction and amplification of the immune response against pancreatic β-cells and, at later stages, to the stabilization and maintenance of insulitis (68). Inflammatory mediators such as cytokines and chemokines probably contribute to the suppression of β-cell function and subsequent apoptosis; they may also inhibit or stimulate β-cell regeneration and might cause peripheral insulin resistance (68). SCFAs have long been known for their anti-inflammatory properties, and this strong effect has been suggested to be dependent on their activation of receptors GPCR41, GPCR43, and GPCR109a, which are required for the normal resolution of inflammation, and/or *via* inhibiting HDACs. The anti-inflammatory role of SCFAs has been well described in different inflammation contexts such as allergic inflammation (69), colitis [summarized in Ref. (67)], and autoimmune diabetes (63). For example, *in vitro* butyrate generates an enhanced production of mRNA for anti-inflammatory IL-18 after culturing in the colonic epithelial cells from neonatal mice; yet, colonic epithelial cells from GPCR109a knockout mice failed to have this upregulating response (70). In addition, involvement of GPCR41 and GPCR43 in the observed anti-inflammatory activity has been suggested (71). These findings together suggest that *via* dampening inflammation, SCFAs may be efficacious in the prevention or treatment of inflammatory diseases such as T1D.

### Molecular Mechanisms Involved in the Beneficial Effects of SCFAs

Short-chain fatty acids maintain the gut integrity *via* two major actions, namely, enhancement of the assembly of TJs and promotion of mucin production by intestinal epithelial cells such as goblet cells. In a Caco-2 cell model, butyrate has been shown to enhance the intestinal barrier by regulating the assembly of TJs *via* activation of AMP-activated protein kinase (64). Correspondingly, in female NOD mice, diet that yielded a large amount of butyrate significantly increased the expression of the TJ-associated protein occludin in the colon (72). Mucins are required for the maintenance of an adequate mucus layer that covers the intestinal epithelium and thereby forms a physical barrier that protects the intestinal epithelium from exposure to antigens. Supplementation of physiological concentrations of butyrate has been shown to increase MUC2 gene expression and MUC2 secretion in both human healthy goblet cell line (24) and colon cancer cell line (73). The beneficial immune-regulatory and anti-inflammatory effects of SCFAs may depend on two major mechanisms. The first involves the activation of various metabolites-sensing GPCR receptors expressed on DCs and intestinal epithelial cells. GPCR43, GPCR41, and GPCR109a are the most well-known GPCRs that contribute to the action related to SCFAs, they can act as receptors with distinct specificity and affinity for each individual SCFA. For example, GPCR43 is activated by SCFAs with varying potency—acetate > propionate > butyrate, while GPCR109a is mainly activated by butyrate. GPCR43 and GPCR109a expression is identified on both DCs and intestinal epithelial cells (74, 75). Interestingly, these two GPCR signaling seem to selectively induce tolerance. In an allergy mouse model, high-fiber feeding has been shown to increase the potency of tolerogenic CD103^+^ DCs in the MLN *via* promoting production of acetate and butyrate and activity of GPCR43 and GPCR109a (75). Importantly, the activation of GPCRs by SCFAs is not limited to the intestinal sites. Both GPCR41 and GPCR43 have been described to be expressed by β-cells (76), activation of these receptors by butyrate in the pancreatic β-cells of male NOD mice has been shown to be responsible for the protective effects against T1D (63).

In addition to binding to GPCRs, SCFAs can act as inhibitors for HDAC, which has genetic association with diabetes and inhibition of which has been demonstrated to promote β-cell development, proliferation, differentiation and function (77). In the NOD mice receiving butyrate- and acetate-yielding diets, the acetate diet has been shown to substantially decrease HDAC3 expression in islet antigen-presenting B cells in the spleen, therefore may lead to a tolerogenic B-cell phenotype and prevent self-antigen-specific T cells proliferation (72). Similarly, in DCs, inhibition of HDACs leads to downregulation of the expression of co-stimulatory markers such as CD86 and CD40, suggesting that butyrate through inhibiting HDAC activity may in turn be able to influence T cells differentiation or function (78).

### Evidence of SCFAs on the Prevention of Autoimmune Diabetes Development

Before reviewing the studies reporting the protection of SCFAs against T1D, it is worth mentioning that the NOD mouse model develops spontaneous diabetes and mimics the situation in the pancreas of patients with respect to the expression of the two major inflammatory cytokines TNF-α and IL-1β and therefore resembles human T1D very well (79). The NOD mouse has therefore been widely used in the investigation of T1D.

By injecting NOD mice with butyrate, researchers demonstrated that butyrate controls pancreatic inflammation *via* governing cathelicidin-related antimicrobial peptide (CRAMP) production by pancreatic β-cells (63). CRAMP has been suggested to harbor positive immune-regulation on pancreatic macrophages and conventional DCs maintaining immune homeostasis in the pancreas *via* Tregs induction. Treatment of NOD mice with butyrate increased pancreatic CRAMP production and stimulation of islet culture, and also increased CRAMP production, indicating a direct effect of butyrate on pancreatic β-cells. The induced CRAMP production was mediated through GPCRs, known to be expressed by pancreatic β-cells (62, 73). Furthermore, the authors showed that deletion of the gut microbiota using antibiotics decreased the pancreatic CRAMP production, indicating the critical role of gut microbiota in the observed protective effects. These data again support that the gut microbiota might regulate the development of T1D *via* SCFAs-dependent pathway.

**Figure 3:**
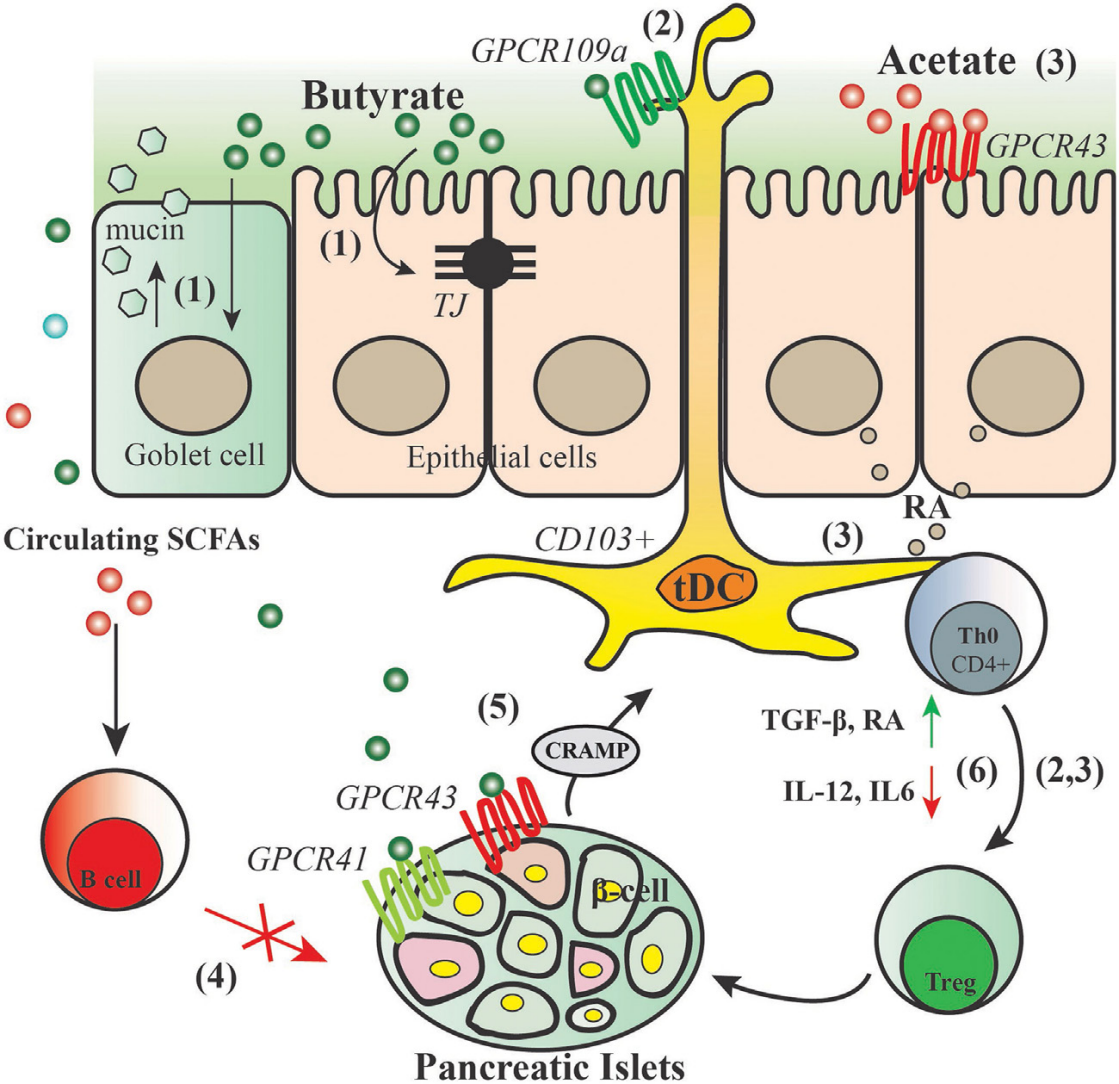
Proposed action for modes of short-chain fatty acids (SCFAs) in the protection of type 1 diabetes. SCFAs generated by gut commensal bacteria in the process of fermentation may have substantial effects on the immune system and intestinal epithelium through different modes of action: (1) butyrate maintains gut integrity by inducing mucin synthesis and TJ production. (2) Butyrate promotes GPCR109a activity, thus inducing CD103^+^ DCs that promote the proliferation of functional regulatory T cells (Tregs). (3) Acetate activates GPCR43 signaling on the intestinal epithelial cells thus promoting their production of retinoic acid (RA), RA leads to increased ALDH activity in mesenteric lymph nodes CD103^+^ DCs and a concomitant increase in regulatory T cells. (4) Acetate reduces marginal zone B cells and therefore reduces expansion of islet-autoantigen-reactive T cell. (5) Butyrate binds to GPCR41 and GPCR43 thus controlling the production of cathelicidin-related antimicrobial peptide (CRAMP) by pancreatic β-cells; CRAMP promotes the induction of pancreatic regulatory DCs and generation of pancreatic FoxP3^+^ Tregs. (6) SCFAs dampen diabetes-related inflammatory cytokines and induce regulatory cytokines and mediators that lead to induction and expansion of Tregs.

In both NOD.Myd88^−/−^ (under SPF and GF conditions) and NOD mice, specialized artificial diets designed to release acetate and butyrate showed a strong inverse correlation between SCFAs concentrations with progression to diabetes (72). Feeding the mice with acetate- or butyrate-releasing diets boosted the concentration of acetate and butyrate in the feces and in hepatic and peripheral blood, respectively. Interestingly, these two diets were shown to have substantial effects on the immune system *via* different mechanisms of action. Acetate-releasing diet was shown to be particularly effective at inhibiting the proliferation of autoimmune T effector cells through decreasing the number of splenic B-cells, which not only have a pathogenic role in autoantibody production and in the transition from insulitis to clinical diabetes but also serve as important APC for islet antigen-reactive T-cells, whereas butyrate-releasing diet functions through promoting the frequency and function of Tregs (72). Taken together, results from these studies support the notion that early-life environmental factors (including the complex mixture of HMOS) govern the interplay between immune system and gut microbiota *via* bacterial metabolites such as SCFAs and thus control the development of autoimmune diseases such as autoimmune diabetes. Figure 3 depicts a model of proposed modes of action by which SCFAs may beneficially regulate the development of T1D.

## What is Known about the Role of Human Milk in the Protection Against T1D?

It is widely accepted that providing infants with human milk is beneficial for development of the newborn. The protective effect of breastfeeding against T1D has been hypothesized since the early 1980s. A recently published nested case–control study indicated that higher human milk consumption was associated with a decreased risk of primary insulin autoimmunity, whereas cow’s milk consumption was associated with an increased risk. This suggests that components of human milk (including fatty acids, HMOS) may contribute to the protective effects against early autoimmunity (80). From birth cohorts (from Norway and Denmark), additional evidence for the fact that human milk may reduce the risk of T1D is provided (81). Furthermore, duration of breastfeeding (whether exclusive or not), use of infant formulas and cow’s milk, as well as the age of introduction of solid food seem to affect the development of T1D (82). For example, a case–control study involving 1,390 preschoolers demonstrated that receiving human milk for more than five months protects against T1D (83). Exposure to gluten and other cereals early in life have been linked with an increased risk of developing T1D collectively with the notification that breastfeeding is suggested to play a role in protection at the time of cereal introduction (84). In a study (MIDA) investigating the relationship between breastfeeding duration with the risk of developing T1D in genetically susceptible newborns, breastfeeding for a duration of 12 months or longer was shown to predict a lower risk for T1D (85). Given the scientific evidence indicated in most published studies, the promotion of breastfeeding should be encouraged in the first year of life to prevent and/or delay the onset of T1D.

It is noteworthy that current evidence regarding the effect of breastfeeding and development of T1D are all derived from observational studies. Little is known about the different components of human milk and their role in T1D development. Human milk provides the infant with a unique and dynamic composition of lipids, proteins, carbohydrates, minerals, and vitamins required for optimal development and protection in early life (86). Next to these macro- and micronutrients, human milk contains multiple functional compounds, including soluble immunoglobulin A, lactoferrin, cytokines, long-chain poly unsaturated fatty acids, hormones, growth factors, oligosaccharides, and beneficial bacteria (9), which support and augment independently or in combination the development of immune system and primes the mucosal interplay with microbiome. It is important to realize that genetic polymorphism in specific genes of mothers determine their HMO composition. A recent study has linked FUT2-dependent HMOS with reduction in allergic disease in breast-fed infants later in life, suggesting a potential protective role of specific HMO compositions against the development of immune disorders such as T1D (87). Furthermore, an interesting implication of the association between FUT2 non-secretor status and the gut microbiome in the pathogenesis of T1D has been provided (88). However, in order to fully understand the relationship between the complex mixture of HMOS and T1D development, large RCTs are required.

## HMOS may Provide a Strategy for Prevention of T1D

The beneficial health effects of human milk cannot be assigned to one single compound; it is generally accepted that HMOS directly and indirectly play a pivotal role in this development. Next to lactose and lipids, HMOS are the third most abundant compound in human milk. More than 200 different structures have been identified, which are unique to human milk. HMOS are a family of complex unconjugated glycans consisting of the monosaccharides glucose, galactose, fucose, *N*-acetylglucosamine, and *N*-acetylneuraminic acid, with the disaccharide lactose at the reducing end. The basic HMO structure can be either fucosylated or sialylated resulting in, respectively, neutral and acidic oligosaccharides. The production of HMOS is regulated by the various glycosyltransferases in the mammary gland. Differences in genetically determined glycosyltransferase pattern affect HMO amount and composition between mothers (89). Besides the inter-individual variation, the concentration and the composition of HMOS in human milk varies over the course of lactation to provide the suckling infant with the optimal nutritional needs over time. Colostrum, first milk secreted at time of parturition, contains approximately 20–25 g/l HMOS, whereas matured human milk declines in HMO concentration to as much as 5–15 g/l (90).

Several research groups (including our own) have demonstrated the preventative and/or therapeutic potential of selected HMOS in immune development, reducing food allergy (91) and infectious episodes (90). However, to the best of our knowledge, there are scarce published reports available on the effects of HMOS in T1D. Given the knowledge that gut microbiota and their metabolites are promising novel targets in the prevention of T1D due to their potential to modulate the mucosal immunity, it seems plausible to assume that HMOS (provided as complex prebiotic oligosaccharide mixture) in early life can be a promising tool to prevent the onset of immune disorders (including T1D) in later life. Based on the preventative role breastfeeding has on the development of T1D, we will discuss in this section the perspectives of HMOS and postulated effects related to autoimmune regulation.

### HMOS Optimize Gut Microbiota Profile

As discussed above, the diminished level of bacterial diversity and abundance of specific bacterial groups have been correlated with the onset and progression of T1D, therefore, proper modification of the gut microbiota seems to provide useful therapeutic strategies for controlling the disease. This notion has been consistently supported by many recent studies, one example is delivery of modified probiotic bacteria, which reset the immune system of the NOD mice toward β cell-specific tolerance and completely prevented T1D (92). The complex mixture of HMOS contributes to building a healthy and stable gut microbiota. The abundance of specific Bifidobacteria has been found to be negatively associated with β-cell autoimmunity in children with two or more diabetes-related autoantibodies in several studies from different countries (18, 93, 94). HMOS were originally described as the “Bifidus factor” in human milk, stimulating the growth of Bifidobacteria (95). Over the past decade, it has been demonstrated that HMOS can act as prebiotics stimulating the growth of distinct bacteria, predominantly mutualists. Prebiotics are defined as “non-digestible food ingredients that beneficially affect the host by selectively stimulating the growth and/or activity of one or limited number of bacteria in the colon, and thus improving host health” (96).

Human milk oligosaccharides stimulate the growth of certain Bifidobacteria and are fermented resulting in the production of metabolites (providing specific SCFA profiles). The mutualist *Bifidobacterium infantis* and to a lesser extend *Bifidobacterium bifidum* have been shown to grow well on HMOS as the only carbohydrate source (97–99). By contrast, the growth of *Bifidobacterium breve* and *Bifidobacterium longum* were moderate on total HMOS; however, *B. breve* showed high levels of growth on the oligosaccharides lacto-*N*-tetraose and lacto-*N*-neotetraose (LNnT) (98, 100). Consistently, a recent study has shown that addition with specific HMOS, 2′-fucosyllactose (2′-FL) into formula led to more abundant *Bifidobacterium* in infants compared with no prebiotic supplemented (101). Regarding the mechanisms whereby those mutualists may benefit the development of T1D, it has been shown that Bifidobacteria, including *B. infantis* and *B. bifidum*, grown on HMOS reduce occludin relocalization and enhance the expression of junction adhesion molecule and occludin in Caco-2 cells. Furthermore, they upregulated the expression of the anti-inflammatory cytokine IL-10, while downregulating the expression of TNF-α (102). These findings suggest the beneficial effect of HMO-grown Bifidobacteria on the gut integrity. The consumption of HMOS by mutualists results in the production of lactate and subsequently SCFAs (butyrate, acetate, and propionate), resulting in significant pH reduction. Furthermore, it has been shown that non-mutualists, such as *C. perifringens* and *E. coli* K12 did not consume HMOS nor produce fermentation products (99). More importantly, the fermentation products such as SCFAs produced by the mutualists not only inhibit the growth of these non-mutualists (103) but also directly or indirectly modulate the immune system, thereby maintaining the gut integrity as described in the previous section. Thus, it is clear that the prebiotic effects of the complex HMOS as well as subsequent bacterial metabolites may play a protective role in T1D. The specific selection and influence of HMOS in encouraging or inhibiting colonization of other specific microbial groups such as Bacteriodetes and Firmicutes in the context of T1D should be investigated in more detail and implemented with great care.

### HMOS May Maintain Intestinal Barrier Function in a Microbiota-Independent Manner

Besides modulating gut integrity indirectly *via* specific microbiota, it can be speculated that HMOS may also have direct effect on intestinal barrier function, based on the current evidence of prebiotic oligosaccharides. Within our own research group, the direct effects of different prebiotic oligosaccharides on the acceleration of the TJ reassembly and reduction of CXCL8 release have been demonstrated in a Caco-2 cell model for intestinal barrier dysfunction (104). An earlier *in vivo* study by us indicated that specific oligosaccharide may maintain gut integrity *via* regulating the expression of CLDN3 (105), which is a TJ protein. These findings suggest that specific prebiotic oligosaccharides can support the maintenance of barrier integrity, although there is currently no evidence regarding the direct, microbiota-independent effects of authentic HMOS on this aspect available. Future research is needed to elucidate and verify the beneficial potential of HMOS regarding maintaining intestinal barrier function, which may contribute to the proposed protective effects of HMOS against T1D.

### HMOS Modulate Systemic and Mucosal Immune System

Although changes in gut microbiota composition and intestinal mucosal environment can indirectly influence the immune system, it is also likely that HMOS may act directly on systemic immune cells since specific HMOS, such as 2′-FL, 3′-sialyllactose, 6′-sialyllactose, and LNnT, have been detected within the intestine and in systemic circulation (106, 107). As discussed above, tDCs function to control effector and regulatory mechanisms relevant to pathology of autoimmune diseases such as T1D (34). DCs express many receptors such as DC-specific intercellular adhesion molecule-3-grabbing non-integrin and TLRs that can recognize the oligosaccharide structures of their glycoprotein ligands. Since some of the HMOS are structurally similar to selectin ligands, it is likely that HMOS can bind directly to DCs and trigger signaling that results in changes to DCs phenotypes and functions, as well as subsequent T cell priming. A recent study showed the immune-modulatory properties of the prebiotic complex mixture—short-chain galacto-oligosaccharides (scGOS) and long-chain fructo-oligosaccharides (lcFOS) in a ratio of 9:1 (mimicking the size and functional characteristics of the complexity of authentic HMOS) on human monocyte-derived DCs, *in vitro* (59). Stimulation of immature DCs with scGOS/lcFOS led to significantly enhanced release of the cytokine IL-10, while no IL-12p70 release was detected. Incubation with the non-digestible oligosaccharides barely upregulated the expression of maturation markers compared to LPS-matured MoDCs. The stimulation of MoDCs with scGOS/lcFOS together with a TLR-4 antagonist effectively abolished MoDC IL-10 release, indicating the potential of these non-digestible oligosaccharides in regulating the immune system. The impact of a complex mixture of HMOS and individual HMOS on DC development should be assessed, which may provide important implications for the potential of HMOS for prevention and treatment of T1D.

## Interplay Between the Mucosal Immune System and Gut Microbiota Development in the Prevention of T1D—An Overview

So far, we reviewed the limited knowledge available on the involvement of the intestinal immune system, including altered microbial composition, leaky gut, and altered mucosal immune response, in the etiology of T1D. Furthermore, we discussed the beneficial effects of human milk, and highlighted specifically the role of SCFAs and the complex mixture of HMOS, on each of these aspects.

**Figure 4:**
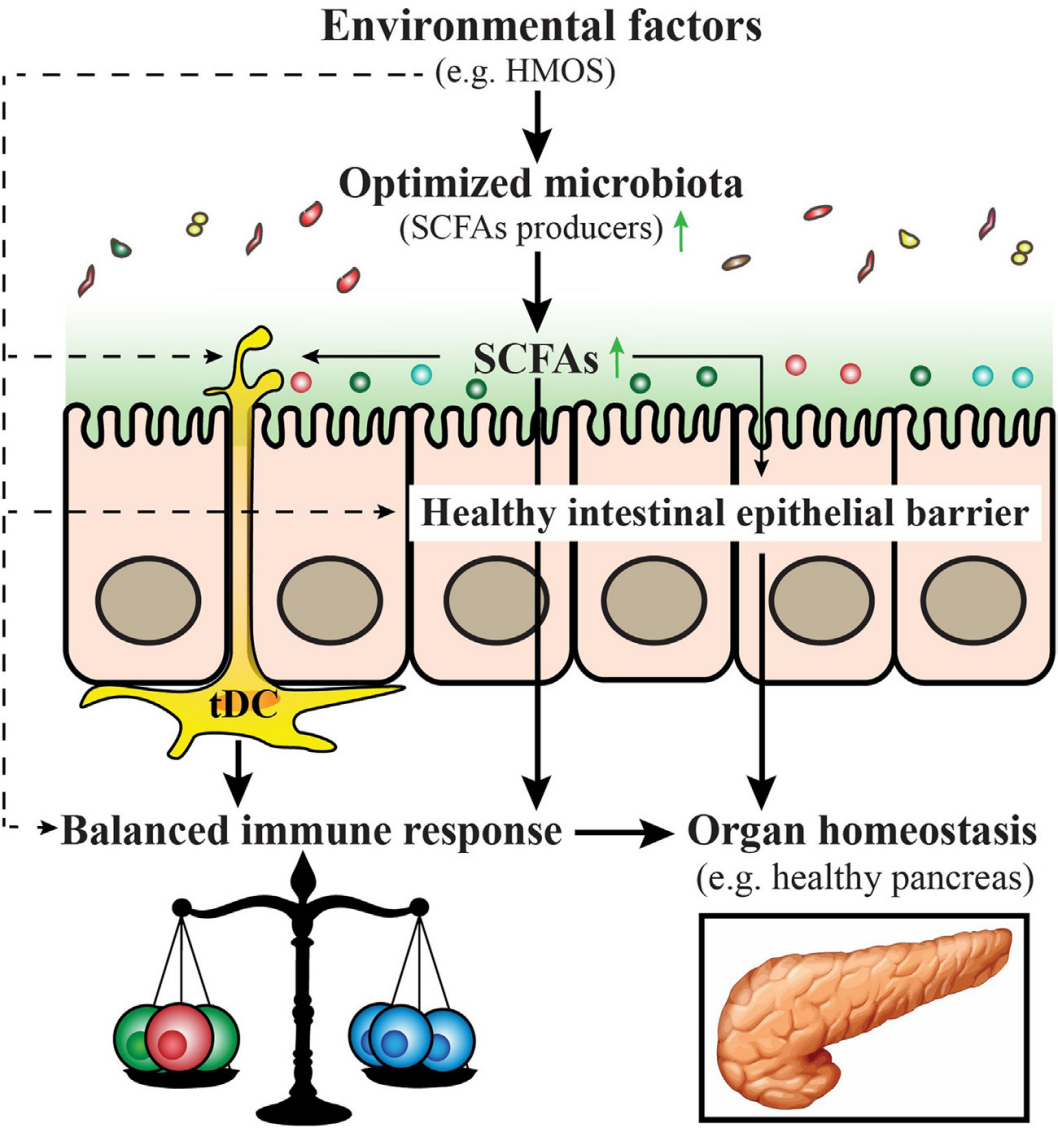
Proposed model for prevention and/or treatment for type 1 diabetes (T1D) by early-life nutritional factors. Early-life nutritional factors such as human milk oligosaccharides (HMOS) can influence the development of T1D *via* direct effects (dash arrows) including induction of an optimized gut microbiota composition with increased short-chain fatty acids (SCFAs) producing bacteria, maintenance of gut integrity, induction of tolerogenic dendritic cells and subsequent balanced T-cell responses; and indirect effects (solid arrows), through effects on the composition of the microbiota, which produce metabolites such as SCFAs that have a favorable regulatory function on immune responses.

A model is provided within a schematic overview (Figure 4) emphasizing the role of early-life environmental factors in the modulation and/or prevention of T1D in early life (Box 1).

Box 1 **An overview of early-life environmental factors involved in type 1 diabetes (T1D) development.**

✓ Development of, and/or cooperation among the intestinal microbiota, the intestinal barrier, and the mucosal immune system are pivotal to mucosal health and are often perturbed in T1D.
✓ Tolerogenic dendritic cells (tDCs) play a critical role in maintaining peripheral and central tolerance in autoimmunity, induction of antigen-specific tDCs offers the possibility to prevent or treat T1D.
✓ Human milk is established to maintain a symbiotic gut microbiota by promoting the growth of beneficial commensal bacteria and inhibiting the adhesion of pathogens and seems to provide tolerogenic factors against development of T1D.
✓ Alterations in the commensal microbiota are associated with human disease, and modulation of the microbiota can modulate disease development.
✓ Short-chain fatty acids (SCFAs) may enhance the epithelial barrier function, induce tDCs, reduce specific B-cells and influence expansion of autoreactive T-cells, control cathelicidin-related antimicrobial peptide production, as well as induce an anti-diabetogenic cytokine profile.
✓ Human milk oligosaccharides intervention in early life is an attractive strategy for the prevention of T1D based on their prebiotic, SCFAs-boosting, microbiota-independent immunomodulatory, and gut microbiota-modulatory effects.

## Future Perspectives

For the last decade, accumulating evidence has demonstrated that the GI tract plays a pivotal role in processes eventually contributing to the destruction of pancreatic β-cells and therefore the development of T1D. However, research focused on the underlying mechanisms unraveling the contribution of human milk is limited. Due to advances in next generation sequencing technologies, studies on the role of the gut microbiota in autoimmune diseases becomes more accessible. To date, however, only two cohort studies studying the microbiome development in T1D, with relatively small size, are available. Clearly, more research is needed to understand the exact role of environmental factors, the microbiota, gut integrity, and mucosal immune system in T1D development. Future research in this field should focus on the interplay at mucosal surfaces, beginning immediately after birth and continuing until T1D onset, to investigate the earliest alterations in these different elements contributing to the development of T1D. Furthermore, additional interest should be paid to the functionality of bacteria associated either with the development or prevention of T1D. Such findings will not only provide understanding on the pathology and development of T1D but will also provide tools and insights in order to prevent the onset of T1D. Notably, the possibility of primary prevention of T1D with a diet-based treatment would be of considerable interest. Increasing amounts of valuable research is being performed on the beneficial effects of HMOS on gut and immunity. Several synthetic oligosaccharides have recently become commercially available with identical structures to the real individual HMOS. However, it is of utmost importance to study the effects of these single HMOS normally present within a complex matrix, on the development of autoimmune disorders such as T1D. It may provide us with important information for mimicking the complex mixture of HMOS regarding anti-diabetogenic benefits. Furthermore, more prospective cohort studies should be performed to form a basic understanding of the relation between breastfeeding and T1D with special attention to the microbial diversity, intestinal and systemic immune-modulatory properties and the cross talk between GI tract and pancreas.

## Author Contributions

LX, BL, and WW have written the review; GF and JG supervised the program; and BS made specific contributions to the program with regard to human milk and in particular functional oligosaccharides. All authors listed have approved it for publication.

## Conflict of Interest Statement

JG is head of the Division of Pharmacology, Utrecht Institute for Pharmaceutical Sciences, Faculty of Science at the Utrecht University, and partly employed by Nutricia Research. Both BS and BL are employed by Nutricia Research. BL, as indicated by the affiliations, is leading a strategic alliance between University Medical Centre Utrecht/Wilhelmina Children’s Hospital and Nutricia Research.

## Abbreviations

2′-FL, 2′-fucosyllactose; 3′-SL, 3′-sialyllactose; 6′-SL, 6′-sialyllactose; APC, antigen-presenting cells; BB-DP, BioBreeding Diabetes Prone; CRAMP, cathelicidin-related antimicrobial peptide; DC, dendritic cell; DC-SIGN, dendritic cell-specific intercellular adhesion molecule-3-grabbing non-integrin; FoxP3, Forkhead box protein 3; FUT 2, fucosyltransferase 2; GAD, glutamic acid decarboxylase; GF, germ-free; GI tract, gastrointestinal tract; GPCR, G-protein-coupled receptor; HAT, histone acetyl transferase; HDAC, histone deacetylase; HIF, hypoxia inducible factor; HMOS, human milk oligosaccharides; JAM, junction adhesion molecule; lcFOS, long-chain fructo-oligosaccharides; LNnT, lacto-*N*-neotetraose; MHC-I (II), major histocompatibility complex class I (II) molecules; NOD, non-obese diabetic; RA, retinoic acid; SCFAs, short-chain fatty acids; scGOS, short-chain galacto-oligosaccharides; SPF, specific pathogen free; T1D, type 1 diabetes; tDC, tolerogenic dendritic cells; Th, T-helper cell; TJ, tight junction; TLRs, toll-like receptors; Tr1, type-1 regulatory T cells; Tregs, regulatory T cells; Thsup, T helper suppressor cells.
